# Guideline‐augmented prompting improves comparative preference and response consistency of large language model outputs for orthopaedic anaesthesia questions: A controlled prompting study

**DOI:** 10.1002/jeo2.70922

**Published:** 2026-10-03

**Authors:** Anita Széll, Yinan Yu, Jacob F. Oeding, Felix C. Oettl, Adam Piasecki, Keti Dalla, Tobias Siöland, Mathias Hård af Segerstad, Fredrik Olsen, Peter Larsson, Stefano Zaffagnini, Kristian Samuelsson, Fredrik Hessulf

**Affiliations:** ^1^ Department of Anesthesiology and Intensive Care Medicine, Institute of Clinical Sciences Sahlgrenska Academy Gothenburg Sweden; ^2^ Department of Anesthesiology and Intensive Care Medicine Sahlgrenska University Hospital Mölndal Mölndal Sweden; ^3^ Department of Computer Science and Engineering Chalmers University of Technology Gothenburg Sweden; ^4^ Department of Orthopaedics, Institute of Clinical Science Sahlgrenska Academy Gothenburg Sweden; ^5^ Department of Anesthesiology and Intensive Care Medicine Sahlgrenska University Hospital Gothenburg Sweden; ^6^ Clinica Ortopedica e Traumatologica II IRCCS Istituto Ortopedico Rizzoli Bologna Italy; ^7^ Dipartimento di Scienze Biomediche eNeuromotorie (DIBINEM) University of Bologna Bologna Italy; ^8^ Department of Orthopaedics Sahlgrenska University Hospital Mölndal Mölndal Sweden

**Keywords:** GPT, large language models (LLMs), orthopaedic anaesthesia, prompt engineering

## Abstract

**Purpose:**

Large language models (LLMs) are increasingly used in clinical contexts; however, performance in complex perioperative decision‐making remains uncertain. Orthopaedic anaesthesia presents a demanding test case due to comorbidity burden and guideline‐dependent management. Whether successive LLM generations and guideline‐augmented prompting improve clinical alignment, comparative performance and response consistency was evaluated in this study.

**Methods:**

In this controlled prompting study, 34 orthopaedic anaesthesia questions spanning seven clinical subdomains were independently answered by three LLMs (GPT‐3.5‐turbo, GPT‐4o and GPT‐5.2) and three expert anesthesiologists. GPT‐4o and GPT‐5.2 each generated responses with and without access to relevant clinical practice guidelines. All responses were anonymized and evaluated in blinded pairwise comparisons by an independent guideline‐informed LLM‐as‐a‐judge (GPT‐5.2) using the same guideline material as the reference standard. The judge recorded preference, response consistency and confidence. Intra‐model consistency was assessed from repeated independent generations of each question.

**Results:**

All LLMs were preferred over human experts in pairwise comparisons without guideline augmentation, with win rates of 67.6% (GPT‐3.5‐turbo), 79.4% (GPT‐4o) and 95.1% (GPT‐5.2). Performance was improved by guideline augmentation, most notably for GPT‐4o (91.2%, +11.8 percentage points), while GPT‐5.2 approached ceiling performance (97.1%). Response consistency varied between models. GPT‐4o showed the highest baseline consistency (80.4%), whereas GPT‐5.2 demonstrated no fully contradictory outputs but showed greater partial variability. Guideline augmentation numerically improved GPT‐5.2 consistency (66.7% to 80.4%) and reduced inter‐model differences. Directed qualitative analysis suggested that reviewer preference for later GPT generations was associated with greater completeness, explicit clinical reasoning and guideline‐oriented responses.

**Conclusion:**

Successive LLM generations demonstrated progressively improved performance in orthopaedic anaesthesia reasoning. Guideline‐augmented prompting enhanced response quality, particularly for intermediate models. Guideline‐informed LLM‐as‐a‐judge evaluation appears promising for comparative assessment but requires further validation.

**Level of Evidence:**

NA.

## Graphical abstract

Guideline‐augmented prompting improved the comparative preference and response consistency of LLM‐generated answers to orthopaedic anesthesia questions, with benefits varying across model generations.

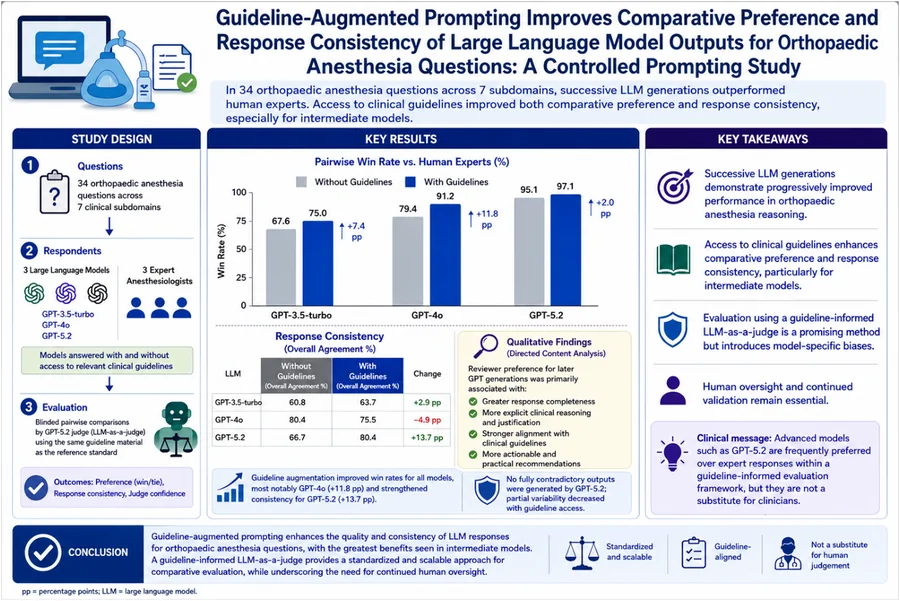

**Abbreviations**
GPT: Generative Pre‐trained Transformer
LLM: large language model

## INTRODUCTION

Large language models (LLMs) are increasingly used in clinical education, documentation, patient communication and orthopaedic research [1, 2, 6, 9, 13, 19, 21]. In orthopaedics, they have generated clinically acceptable responses to patient questions, although variability in completeness, consistency and clinical accuracy remains a concern [15, 16, 20]. Similar heterogeneity has been reported in critical care and anaesthesiology, where differences in appropriateness and agreement with clinician recommendations have been observed across LLM generations [3, 5]. These findings highlight the need for standardized evaluation frameworks [18].

Perioperative orthopaedic anaesthesia represents a challenging domain for LLM assessment because anaesthetic decisions require integration of patient comorbidities, anticoagulation therapy and procedure‐specific considerations [10]. As LLMs become increasingly incorporated into clinical education and decision support, understanding how performance evolves across successive model generations is essential.

Prompt engineering, particularly the incorporation of clinical guidelines, may reduce response variability and improve alignment with evidence‐based practice [4, 17]. Guideline‐informed evaluation frameworks, in which an LLM with access to relevant guidelines compares candidate responses, provide a standardized method for assessing clinical reasoning and comparing model generations [12, 27]. However, this approach has not been systematically evaluated in orthopaedic anaesthesia.

The aim of this study was to compare GPT‐3.5‐turbo, GPT‐4o and GPT‐5.2 with expert anesthesiologists in answering orthopaedic anaesthesia questions. We hypothesized that newer GPT generations would outperform earlier models and demonstrate closer alignment with guideline‐informed clinical reasoning than expert clinician responses.

## METHODS

### Study design

The pipeline evaluates LLM performance on 34 clinical questions pertaining to orthopaedic anaesthesiology (Appendix S1) and spanning across 7 subdomains (hip fracture, knee surgery, regional anaesthesia, PONV, bone cement implantation syndrome, general anaesthesia and post‐operative pain). Questions 1–22 are mapped to one or more of nine published clinical practice guidelines. Questions 23–27 did not have a corresponding guideline. Questions 28–34 were mapped to a current review article. For a list of guideline and review references, see Appendix S2.

### Answer generation

Three LLMs, GPT‐3.5‐turbo (gpt‐3.5‐turbo‐0125), GPT‐4o (gpt‐4o‐2024‐08‐06) and GPT‐5.2 (gpt‐5.2‐2025‐12‐11), answered each question under two prompting conditions: without supplied guideline material, and with the relevant guideline excerpts provided in‐context. The with‐guidelines condition was run for GPT‐4o and GPT‐5.2. For each combination of question, model and condition, the model was sampled three times to support the self‐consistency analysis.

### Model‐versus‐expert evaluation

Three board‐certified anaesthesiology experts (10–20 years of experience, extensive experience in orthopaedic anaesthesia, no access to guidelines) independently provided free‐text answers to each question using the same instructions and response‐length constraints as the LLMs. Responses were evaluated in their original form without language editing or correction to preserve semantic content. The experts were blinded to the study hypothesis. All pairwise comparisons were judged by GPT‐5.2 supplied with relevant guidelines. In every comparison, the order of the two answers shown to the judge was randomized via a swap flag, and all tabulations use the labels actually shown to the judge. The judge received the relevant guideline excerpts as reference and was instructed to prefer the answer that was more correct, safe and clinically actionable, penalizing contradictions with the guidelines. Each model answer was paired with each of the three expert answers, and the judge returned a winner (A, B or Tie); no ties were returned. The unit of independent observation is the question (*n* = 34); the three expert ratings attached to the same question share the same model output and are not independent observations of the model. For every (model, condition and expert) cell, we report the win count over 34 questions and a 95% Wilson confidence interval on the win rate.

### Self‐consistency evaluation

Using the same judge, swap randomization, guideline reference and instructions as in the model‐versus‐expert evaluation, we ran a second judging pass: for each (question, model and condition), the three independent model samples were compared in all three pairwise combinations, and the judge returned a 0/1/2 consistency score (2 = consistent, 1 = partially consistent and 0 = inconsistent). The unit of independent observation remains the question; the three repeat‐pair ratings attached to the same question share the same model samples. Per (model and condition), we report the proportion of each score and the mean score; for each quantity we first aggregate the three repeat‐pair ratings within each question to a per‐question mean, then average those per‐question means across the 34 questions, with 95% intervals from a non‐parametric cluster bootstrap of 20,000 resamples that resample questions with replacement.

For the effect of providing guidelines, per‐question paired differences in mean consistency score were computed within each model. The mean of the 34 differences is reported with a cluster‐bootstrap 95% interval. A two‐sided Wilcoxon signed‐rank test was performed with zeros dropped and the exact distribution applied to the remaining ranks. Because the differences take only a small number of discrete values and ties are common, a two‐sided exact sign test on the non‐zero differences is reported as a tie‐robust supplement.

### Position‐bias diagnostic

The position‐bias diagnostic uses the self‐consistency comparisons, in which both sides of every judgement are independent samples from the same model under the same condition and are exchangeable in expectation, so any A versus B asymmetry in the judge's decisive verdicts is attributable to position rather than content. Per (model and condition), we report counts of A‐wins, B‐wins and ties and the share of A‐wins among decisive verdicts. We do not report a formal test because the three repeat‐pair ratings per question are not independent observations of the position effect.

### Directed qualitative content analysis

To complement the quantitative comparisons, a directed qualitative content analysis [14] was performed on a purposive sample of five clinical questions selected to represent diverse orthopaedic anaesthesia domains (hip fracture, perioperative warming, regional anaesthesia, perioperative pharmacology and post‐operative analgesia). For each question, the three clinician responses and three LLM responses were independently evaluated by two orthopaedic anaesthesia reviewers using a structured workbook. Reviewers assessed guideline concordance, clinically important omissions, potentially unsafe recommendations, strengths and weaknesses for each anonymized response before identifying recurring characteristics. Following completion of the blinded assessments, responses were unblinded, and reviewer comments were synthesized to identify recurring characteristics distinguishing clinician‐ and LLM‐generated responses. The qualitative analysis was intended to explain the quantitative findings rather than provide statistical comparison.

Figure 1 presents the study design flowchart.

**Figure 1:**
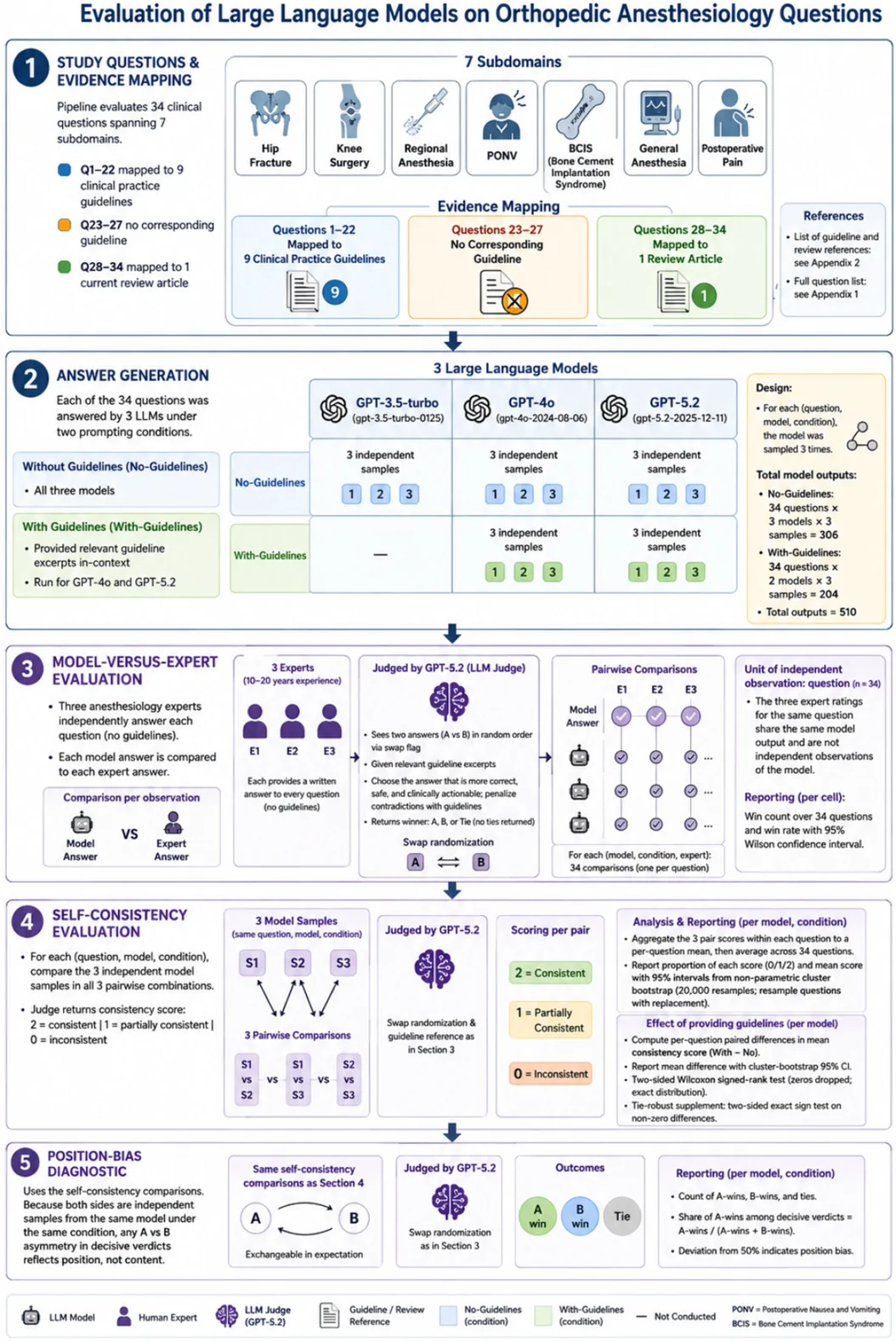
Study design flowchart (generated by GPT 5.5 based on text prompt).

## RESULTS

### Model‐versus‐expert preference

The judge preferred the model answer over the expert answer in every (model, condition and expert) cell, and win rates rose monotonically with model capability across the three experts (Table 1). Adding guideline excerpts in‐context raised GPT‐4o into the range previously occupied by GPT‐5.2, and brought GPT‐5.2 to 33/34 against all three experts, consistent with a ceiling on this question set.

**Table 1:** Win rate of model over expert per (model, condition and expert).

Condition	Model	Expert	Wins/*N*	Win rate [95% CI]
Without guidelines	gpt‐3.5‐turbo	human_1	25/34	73.5% [56.9–85.4]
Without guidelines	gpt‐3.5‐turbo	human_2	21/34	61.8% [45.0–76.1]
Without guidelines	gpt‐3.5‐turbo	human_3	23/34	67.6% [50.8–80.9]
Without guidelines	gpt‐4o	human_1	29/34	85.3% [69.9–93.6]
Without guidelines	gpt‐4o	human_2	25/34	73.5% [56.9–85.4]
Without guidelines	gpt‐4o	human_3	27/34	79.4% [63.2–89.7]
Without guidelines	gpt‐5.2	human_1	33/34	97.1% [85.1–99.5]
Without guidelines	gpt‐5.2	human_2	33/34	97.1% [85.1–99.5]
Without guidelines	gpt‐5.2	human_3	31/34	91.2% [77.0–97.0]
With guidelines	gpt‐4o	human_1	33/34	97.1% [85.1–99.5]
With guidelines	gpt‐4o	human_2	32/34	94.1% [80.9–98.4]
With guidelines	gpt‐4o	human_3	28/34	82.4% [66.5–91.7]
With guidelines	gpt‐5.2	human_1	33/34	97.1% [85.1–99.5]
With guidelines	gpt‐5.2	human_2	33/34	97.1% [85.1–99.5]
With guidelines	gpt‐5.2	human_3	33/34	97.1% [85.1–99.5]

*Note*: Each cell is the proportion of 34 questions on which the judge preferred the model's answer to that expert's answer; 95% Wilson confidence intervals (95% CIs).

### Self‐consistency

GPT‐3.5‐turbo was the least self‐consistent across repeated samples; GPT‐4o and GPT‐5.2 were more consistent and indistinguishable from each other within the bootstrap intervals (Table 2). Adding guidelines did not separate the two newer models on this metric.

**Table 2:** Self‐consistency across three independent samples per question.

Condition	Model	%2 [95% CI]	%1 [95% CI]	%0 [95% CI]	Mean [95% CI]
Without guidelines	gpt‐3.5‐turbo	62.7% [51.0–74.5]	26.5% [16.7–37.3]	10.8% [3.9–18.6]	1.52 [1.34–1.69]
Without guidelines	gpt‐4o	80.4% [70.6–89.2]	17.6% [9.8–26.5]	2.0% [0.0–4.9]	1.78 [1.68–1.89]
Without guidelines	gpt‐5.2	66.7% [52.9–79.4]	33.3% [20.6–47.1]	0.0% [0.0–0.0]	1.67 [1.53–1.79]
With guidelines	gpt‐4o	78.4% [67.6–88.2]	17.6% [8.8–27.5]	3.9% [0.0–9.8]	1.75 [1.61–1.87]
With guidelines	gpt‐5.2	80.4% [70.6–89.2]	16.7% [8.8–25.5]	2.9% [0.0–7.8]	1.77 [1.65–1.88]

*Note*: Distribution of the judge's 0/1/2 consistency score and the mean score per (model, condition); proportions and means are reported with cluster‐bootstrap 95% confidence intervals (95% CIs) by question.

### Effect of guidelines on self‐consistency

The within‐question paired contrast was small in magnitude and opposite in sign for the two models, with 95% intervals that included zero and non‐significant Wilcoxon and exact sign tests in both cases (Table 3). At *n* = 34 questions, we find no evidence that in‐context guidelines change repeat‐sample consistency in either direction.

**Table 3:** Effect of providing guidelines on self‐consistency.

Model	Paired *N*	Difference [95% CI]	Wilcoxon *p*	Sign *p*
gpt‐4o	34	−0.039 [−0.196, +0.118]	0.712	1.000
gpt‐5.2	34	+0.108 [−0.059, +0.275]	0.147	0.383

*Note*: Per‐question paired difference in mean consistency score, with‐guidelines minus without‐guidelines. Cluster‐bootstrap 95% confidence interval (95% CI) on the mean difference; Wilcoxon signed‐rank and exact sign test *p* values on the per‐question differences.

### Judge position bias

In the self‐consistency comparisons, both sides of every judgment are exchangeable samples from the same model, so any A‐versus‐B asymmetry reflects position rather than content. The judge consistently favoured the answer shown in position B across all (model and condition) cells, and issued decisive verdicts more often when guidelines were provided even though the inputs were exchangeable (Table 4). Because each model‐versus‐expert pair in Table 1 was judged in only one orientation, this position effect is not adjusted out of those win rates.

**Table 4:** Judge position‐bias diagnostic, derived from the self‐consistency comparisons in which both sides of every judgment are exchangeable samples from the same model.

Condition	Model	A/B/Tie/*N*	%A among decisive
Without guidelines	gpt‐3.5‐turbo	13/25/64/102	34.2%
Without guidelines	gpt‐4o	9/29/64/102	23.7%
Without guidelines	gpt‐5.2	21/29/52/102	42.0%
With guidelines	gpt‐4o	22/44/36/102	33.3%
With guidelines	gpt‐5.2	25/52/25/102	32.5%

*Note*: Counts of A‐wins, B‐wins and ties per (model, condition) and share of A‐wins among decisive verdicts.

Directed qualitative analysis identified consistent differences in the characteristics of clinician‐ and LLM‐generated responses. Reviewer comments indicated that clinician responses were generally concise and reflected clinical experience but demonstrated greater inter‐individual variability and more frequent omission of guideline‐specific details, patient stratification, monitoring recommendations and supporting rationale. In contrast, LLM responses were consistently described as more structured, comprehensive and guideline‐oriented. Across successive GPT generations, reviewers noted progressive improvements in completeness, explicit clinical reasoning, safety emphasis and practical actionability. Reviewer criticism also differed between groups. Clinician responses were most commonly criticized for omissions and variability, whereas LLM responses were more frequently criticized for insufficient nuance or incomplete interpretation of specific guideline recommendations.

## DISCUSSION

The principal findings of this study are fivefold. First, all evaluated LLMs were preferred over human expert responses in pairwise comparisons, with performance improving across successive model generations. Second, guideline augmentation significantly improved model performance, particularly for GPT‐4o, while GPT‐5.2 demonstrated near‐ceiling performance regardless of augmentation. Third, response consistency varied across models, with guideline augmentation improving stability for GPT‐5.2 but having minimal effect on GPT‐4o. Fourth, qualitative analysis suggested that the greater comparative preference for later GPT generations was primarily associated with increasingly comprehensive, structured and guideline‐oriented responses, rather than isolated differences in factual correctness. Fifth, a guideline‐informed LLM‐as‐judge framework enabled structured evaluation of clinical alignment, highlighting both the strengths and limitations of current LLM performance in orthopaedic anaesthesia contexts.

With respect to comparative performance, all models were preferred over human experts in the majority of pairwise evaluations, with win rates of 67.6% for GPT‐3.5‐turbo, 79.4% for GPT‐4o and 95.1% for GPT‐5.2 without guideline augmentation. These findings support the hypothesis that newer model generations demonstrate improved clinical reasoning and alignment. Notably, GPT‐5.2 achieved consistently high performance across all expert comparisons (91.2–97.1%), suggesting that advanced models are capable of producing responses that align closely with structured clinical expectations. This extends prior work demonstrating acceptable LLM performance in orthopaedics into the more complex domain of perioperative anaesthesia decision‐making [8, 16]. However, because evaluation was conducted using a guideline‐informed LLM‐as‐judge framework rather than direct scoring against guidelines, these results reflect comparative preference and alignment rather than definitive clinical correctness.

Guideline augmentation had a measurable and differential impact across models. GPT‐4o demonstrated a substantial improvement in win rate against human experts (79.4–91.2%, +11.8 percentage points), whereas GPT‐5.2 showed only a modest increase (95.1–97.1%). This pattern suggests that structured contextual prompting can partially compensate for differences in baseline model capability. The limited improvement observed for GPT‐5.2 likely reflects a ceiling effect, as its baseline performance was already high. These findings are consistent with the broader literature on prompt engineering, which demonstrates that incorporating structured clinical context or guidelines can improve alignment with evidence‐based practice [4, 12, 17]. In anaesthesia, where recommendations are often tightly coupled to formal guidelines, such augmentation may serve as a practical mechanism to improve output reliability without modifying the underlying model.

Response consistency analysis revealed important differences in model stability. Without guidelines, GPT‐4o demonstrated the highest full consistency rate (80.4%) and mean consistency score (1.78), whereas GPT‐5.2 showed no fully inconsistent responses but a higher proportion of partially consistent outputs, resulting in a lower full consistency rate (66.7%). This suggests that more advanced models may generate a broader range of acceptable but variably phrased answers rather than strictly identical outputs. With guideline augmentation, GPT‐5.2 consistency improved substantially (full consistency rate 66.7–80.4%), while GPT‐4o remained largely unchanged. The convergence of consistency metrics between models with guideline augmentation indicates that external reference material may help standardize outputs and reduce variability. From a clinical perspective, improved consistency may enhance trust and usability, particularly in settings where reproducibility of recommendations is important.

The use of a guideline‐informed LLM‐as‐a‐judge framework represents a key methodological contribution of this study. By incorporating guideline‐informed evaluation criteria and blinded pairwise comparisons, this approach enables scalable and structured assessment of clinical responses. However, LLM judges do not eliminate bias but transform it; unlike human judges, who are susceptible to cognitive and inter‐observer biases, LLM judges may exhibit training‐data, self‐preference and position biases [11, 22, 23, 24, 26]. To reduce reliance on the model's internal knowledge, the judge was provided with the relevant clinical guidelines as the reference standard. Recent studies have shown that reference‐grounded or guideline‐augmented LLM judges improve agreement with expert evaluation and produce more robust model rankings [7, 12, 25, 27]. Consistent with this, our qualitative analysis suggested that later GPT generations were preferred because their responses were more comprehensive, explicitly reasoned and guideline‐oriented, whereas clinician responses demonstrated greater variability. Nevertheless, guideline‐augmented LLM judges should be regarded as a standardized evaluation tool rather than a replacement for independent human assessment.

Summarizing, these results demonstrate that LLM performance in orthopaedic anaesthesia is influenced by both model capability and prompt design. Advanced models such as GPT‐5.2 can achieve high levels of alignment with clinician responses, and guideline augmentation further improves performance and consistency, particularly for intermediate models. These findings support the potential role of LLMs in perioperative education, while reinforcing the importance of structured prompting and external validation.

### Limitations

Several limitations warrant consideration. First, evaluation was based on pairwise judgements from a guideline‐informed LLM judge rather than direct comparison to objective ground‐truth answers or independent human adjudication. Although providing the judge with relevant clinical guidelines was intended to improve standardization and reduce reliance on the model's internal knowledge, model‐specific biases such as training‐data, self‐preference and position bias may still have influenced the evaluations. In addition, some reference guidelines did not explicitly address every component of the mapped clinical question, requiring interpretation of broader recommendations rather than direct comparison with an explicit reference standard. Second, only three anaesthesiology experts were used as the human reference standard, potentially limiting generalizability. Third, the study included 34 questions, which may not capture the full complexity of orthopaedic anaesthesia practice. Fourth, responses were generated in a controlled experimental setting and may differ in real‐world clinical deployment. Fifth, the directed qualitative analysis was performed on a purposive sample of five questions and was intended to explain the quantitative findings rather than provide statistically generalizable comparisons. Finally, restricting responses to two to three sentences may have disadvantaged the human experts compared to the LLM.

## CONCLUSION

Successive generations of LLMs demonstrate progressively improved performance in answering orthopaedic anaesthesia questions, with advanced models consistently preferred over human experts in pairwise comparisons under a guideline‐informed evaluation framework. Guideline augmentation enhances performance and consistency, particularly for intermediate models and helps standardize outputs across repeated generations. Structured prompting and evaluation frameworks represent important tools for optimizing LLM behaviour in clinical contexts, although human oversight remains essential. Using a guideline‐informed LLM‐as‐a‐judge to compare LLM and clinician performance provides a standardized and scalable framework for comparing clinician and LLM responses but requires further validation against independent human evaluation.

## AUTHOR CONTRIBUTIONS

All listed authors have contributed substantially to this work. Anita Széll, Yinan Yu, Jacob F. Oedling, Felix Oetl, Adam Piasecki, Keti Dalla, Mathias Hård af Segerstad, Fredrik Olsen, Peter Larsson, Kristian Samuelsson and Fredrik Hessulf assisted with the study design, evaluation and interpretation of the results, as well as editing the manuscript. All authors have read and approved the final manuscript.

## FUNDING INFORMATION

The authors have nothing to report.

## CONFLICT OF INTEREST STATEMENT

Kristian Samuelsson is a member of the Board of Directors of Getinge AB (Publ) and a Medtech advisor to Carl Bennet AB. The other authors declare no conflicts of interest.

## ETHICS STATEMENT

The authors have nothing to report.

## Supporting information

Attachment 1. Prompting Questions; Attachment 2. Reference list to referenced guidelines and review articles.

## Data Availability

Data are available upon reasonable request to the corresponding author.
