# Supplementary material for: Guideline‐augmented prompting improves comparative preference and response consistency of large language model outputs for orthopaedic anaesthesia questions: A controlled prompting study
Source: J Exp Orthop. 2026 Oct 3;13(4):e70922. doi: 10.1002/jeo2.70922 (PMC13633554; doi:10.1002/jeo2.70922)
Supplement: Supplementary file 1 [file JEO2-13-e70922-s001.docx]

Supplementary file

Attachement 1. Prompting Questions

Hip fracture

1. What is the recommended transfusion threshold for a geriatric patient awaiting

surgery for a hip fracture?

2. In a patient with a Hip fracture, under what circumstances is it appropriate to

delay surgery beyond 36 hours from admission?

3. How can we optimize perioperative pain relief in patients with hip fracture awaiting

surgery?

4. What additives can be used in addition to common local anesthetics such as

bupivacaine when giving a spinal anesthetic for an elective hip replacement? Give

me three and motivate.

5. In which conditions is High Flow Nasal Cannula recommended during

arthroplasty surgery?

6. Motivate if non-operative management of a hip fracture can be preferred to

operative management?

7. How can hypothermia during and after hip fracture surgery be prevented and

treated?

8. What is the gold standard anaesthesia technique for relocation/reduction of a

dislocated hip after total hip arthroplasty?

Knee surgery

9. Give me the three preferred regional nerve blocks and a one sentence

motivation when managing a total knee arthroplasty if you want to preserve motor

function following the nerve block?

10. What is the advantage of injecting LA in the joint during the arthroscopy for ACL,

compared to peripheral nerve blocks?

11. What anesthesiological alternatives (name 3 with rank) can we use for pain

relief following ACL surgery exists excluding intravenous pain medications?

Regional Anesthesia

12. What are the advantages of using US guided regional anesthesia compared to

using a nerve stimulator?

13. What is the recommended time from last drug intake to high risk regional

anesthesia ( spinal, epidural) for the three most common NOACs?

14. How can apixaban concentrations be used to optimize time to neuraxial block?

15. How do you manage a patient on dual antiplatelet therapy who requires urgent

surgical repair of a HIP fracture?

16. How do you minimize the risk of local anesthetic systemic toxicity (LAST) during

peripheral nerve blocks?

17. Should nerve blocks targeting the brachial plexus (axillary or supraclavicular

nerve block) be combined with a more distal nerve block (targeting the ulnar, radial

and median nerve at the level of the elbow)? Motivate

18. Is there an increased risk of a new shoulder displacement if a regional nerve

block is used to facilitate reduction of a displaced shoulder?

19. During the administration of a spinal anesthetic, does speed of injection affect

the distribution of nerve block and the hemodynamic side effects?

PONV ( Postoperative nausea vomiting)

20. What are the four most important risk factors for PONV in adults undergoing

orthopedic surgery?

21. Which non pharmacological strategies exist to prevent and to treat PONV?

22. What are the pharmacologic choices for treatment of PONV?

Bone cement implantation syndrome ( BCIS)

23. What is the recommended intraoperative approach for preventing and managing

BONE CEMENT IMPLANTATION SYNDROME?

General anesthesia

24. What are the indications and dosage of intravenous dexamethasone in

orthopedic surgery?

25. What is the gold standard anaesthesia technique for the relocation/reduction

of a dislocated shoulder?

26. Is low dose suxamethonium to facilitate reduction of a displaced hip following THA

without a secured airway with an endotracheal tube associated with increased rates of

aspiration?

27. Is there evidence for non-invasive ventilation (such as BiLevel or CPAP) to

prevent complications in the PACU following orthopedic surgery?

Postoperative pain

28. What are the indications for using intrathecal opioids in patients undergoing

spinal anesthesia for lower extremity orthopedic surgery?

29. Among patients undergoing orthopedic surgery with increased risk for

compartment syndrome. Motivate if and how central nerve blocks can be used for

postoperative pain management?

30. What are the advantages and disadvantages of using ropivacaine versus

chirocaine for peripheral nerve blocks in fracture surgery?

31. NSAIDs are commonly used to treat and prevent pain following surgery - Is there

any evidence that short using (5-7 days) COX2 inhibitors have negative effects on

bone healing?

32. Is there any advantage using continuous peripheral nerve block versus single

dose peripheral block with long lasting effects in orthopedic surgery?

33. How should post dural headache be managed?

34. How can the risk of rebound pain be mitigated following a nerve block in

orthopedic surgery?

Attachment 2. Reference list to referenced guidelines and review articles

1.	Griffiths R, Babu S, Dixon P, Freeman N, Hurford D, Kelleher E, et al. Guideline for the management of hip fractures 2020: guideline by the Association of Anaesthetists. *Anaesthesia*. 2021;76(2):225-237. doi:10.1111/anae.15291.

2.	American Academy of Orthopaedic Surgeons. *Management of Hip Fractures in Older Adults: Evidence-Based Clinical Practice Guideline*. Rosemont (IL): AAOS; 2021.

3.	Griffiths R, White SM, Moppett IK, Parker MJ, Chesser TJS, Costa ML, et al. Safety guideline: reducing the risk from cemented hemiarthroplasty for hip fracture 2015. *Anaesthesia*. 2015;70(5):623-626. doi:10.1111/anae.13036.

4.	Donaldson AJ, Thomson HE, Harper NJN, Kenny NW. Bone cement implantation syndrome. *Br J Anaesth*. 2009;102(1):12-22. doi:10.1093/bja/aen328.

5.	Neal JM, Brull R, Horn JL, Liu SS, McCartney CJL, Perlas A, et al. The second American Society of Regional Anesthesia and Pain Medicine evidence-based medicine assessment of ultrasound-guided regional anesthesia: executive summary. *Reg Anesth Pain Med*. 2016;41(2):181-194. doi:10.1097/AAP.0000000000000331.

6.	Gan TJ, Belani KG, Bergese S, Chung F, Diemunsch P, Habib AS, et al. Fourth consensus guidelines for the management of postoperative nausea and vomiting. *Anesth Analg*. 2020;131(2):411-448. doi:10.1213/ANE.0000000000004833.

7.	Kietaibl S, Ferrandis R, Godier A, Llau JV, Samama CM, Schlimp CJ, et al. Regional anaesthesia in patients on antithrombotic drugs: Joint ESAIC/ESRA guidelines. *Eur J Anaesthesiol*. 2022;39(2):100-132. doi:10.1097/EJA.0000000000001600.

8.	Kozek-Langenecker SA, Ahmed AB, Afshari A, Albaladejo P, Aldecoa C, Barauskas G, et al. Management of severe perioperative bleeding: guidelines from the European Society of Anaesthesiology and Intensive Care. *Eur J Anaesthesiol*. 2023;40(4):226-304. doi:10.1097/EJA.0000000000001804.

9. Elkassabany NM, Antosh S, Ahmed M, Nelson C, Israelite C, Mariano ER. A guide to regional analgesia for total knee arthroplasty. *Best Pract Res Clin Anaesthesiol*. 2019;33(1):111-123.

10. Wick EC, Grant MC, Wu CL. Postoperative multimodal analgesia pain management with nonopioid analgesics and techniques: a review. *JAMA Surg*. 2017;152(7):691-697. doi:10.1001/jamasurg.2017.0898.
